# Treatment of Fournier's Gangrene with Combination of Vacuum-Assisted Closure Therapy, Hyperbaric Oxygen Therapy, and Protective Colostomy

**DOI:** 10.1155/2011/430983

**Published:** 2012-01-23

**Authors:** Giovanni Zagli, Giovanni Cianchi, Sara Degl'Innocenti, Jessyca Parodo, Lorenzo Bonetti, Paolo Prosperi, Adriano Peris

**Affiliations:** ^1^Anaesthesia and Intensive Care Unit of Emergency Department, Careggi Teaching Hospital, Largo Brambilla 3, 50134 Florence, Italy; ^2^Department of General Surgery, Careggi Teaching Hospital, Largo Brambilla 3, 50134 Florence, Italy

## Abstract

Fournier's gangrene is a rare process which affects soft tissue in the genital and perirectal area. It can also progress to all different stages of sepsis, and abdominal compartment syndrome can be one of its complications. Two patients in septic shock due to Fournier gangrene were admitted to the Intensive Care Unit of Emergency Department. In both cases, infection started from the scrotum and the necrosis quickly involved genitals, perineal, and inguinal regions. Patients were treated with surgical debridement, protective colostomy, hyperbaric oxygen therapy, and broad-spectrum antibacterial chemotherapy. Vacuum-assisted closure (VAC) therapy was applied to the wound with the aim to clean, decontaminate, and avoid abdominal compartmental syndrome development. Both patients survived and were discharged from Intensive Care Unit after hyperbaric oxygen therapy cycles and abdominal closure.

## 1. Introduction

Fournier's gangrene is a rare necrotizing fasciitis of the perineal, genital, or perianal regions [1]. It is characterized by obliterative endarteritis and thrombosis of the subcutaneous arteries resulting in gangrene of the subcutaneous tissue and overlying skin. Tissue damage may extend to the penis, anterior abdominal wall, buttocks, or thighs [2, 3]. Fournier's gangrene usually starts with perianal or perineal pain, which is often disproportionate to the physical finding such as swelling or pruritus in the affected area. The disease is not limited to young individuals, nor is it limited to men [4]. In most cases, Fournier's gangrene is a polymicrobial, synergistic, and necrotizing infection of the perineal subcutaneous fascia and male genitalia that originates from the skin, urethra, or rectum. The most commonly found microorganisms are E. coli, Bacteroides, Staphylococcus, Proteus, Streptococcus, Pseudomonas, and Enterococcus [5–7].

Patients affected may present fever, malaise for a few days, nonspecific abdominal pain, and general symptoms of infections without any specific symptoms from the perineal area. Depending upon the degree of progression, the skin may look normal, red, or shiny in appearance or may show evidence of ecchymosis, crepitus, or gangrene [6–8]. Advanced age (over 50 years old), obesity, diabetes mellitus, peripheral vascular disease, local trauma, urethral stricture, malignant, and perianal disease have been reported as main predisposing factors [9]. The disease can no longer be considered to be idiopathic, as reported in Fournier's original article [10]. In most of cases, an urologic, colorectal, or cutaneous source can be identified [11]. The disease is no longer prevalent in the younger members of the population, and an analysis clearly revealed a male predominance and a mean age over 50 years [10, 11].

There is a worldwide consensus that immediate radical excision of the gangrene should be accompanied by intensive care measures in patients with Fournier's Gangrene. Treatment must include the application of sepsis management guidelines [12], and, where available, hyperbaric oxygen therapy is highly recommended [13].

Here we report two cases of septic shock due to Fournier's gangrene which recovered well, treated with surgical debridement, hyperbaric oxygen therapy, and vacuum-assisted closure (VAC) therapy.

## 2. Case Presentation

The patients described here were both admitted to the Intensive Care Unit (ICU) of the Referral Center for Hyperbaric Oxygen Therapy (Careggi Teaching Hospital, Florence, Italy). The patients' data were recorded in our ICU-database (FileMaker Pro 5.5v2, FileMaker, Inc, USA). For each patient, demographic, clinical characteristics and laboratory parameters were collected (Table 1). Written, informed consent was obtained from the patients for publication and accompanying images.

During ICU stay, intraabdominal pressure was monitored by using a urinary bladder pressure gauge (AbViser, Wolfe Tory Medical Inc., Salt Lake City, Utah) in supine patients; every 4 hours, 50 mL of saline solution was instilled in the urinary bladder closed system. VAC device used (KCI, San Antonio, TX, USA) is a polyurethane sponge cut to the appropriate size placed over the wound. The sponge, with an 18-F suction tube, is covered with second sterile adherent occlusive dressing. Suction is applied to the sponge using a portable pump. The dressing needs to be changed every 24–72 hours.

### 2.1. Case  1

**Figure 1:**
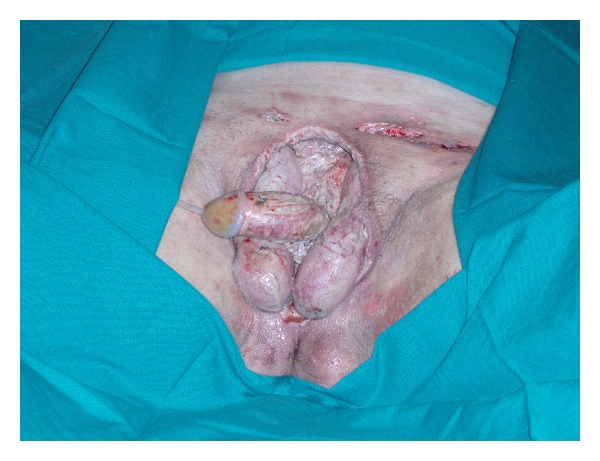


**Figure 2:**
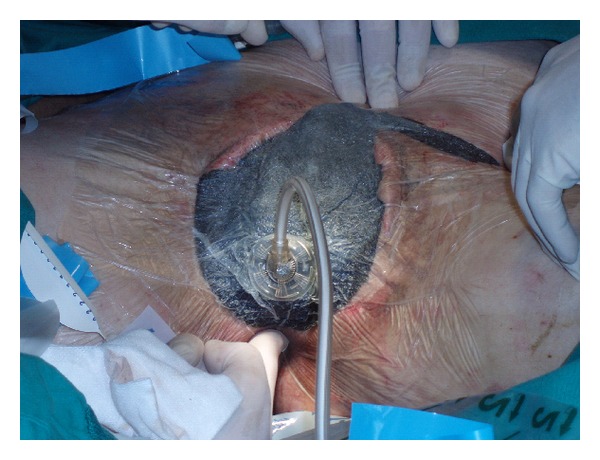


A 63-year-old man was transferred to our ICU from a peripheral hospital with a diagnosis of Fournier's gangrene. Initial symptoms had started one week before patient presentation at the peripheral emergency department. At our ICU admission, the patient was in septic shock, and infection involved all the scrotum, genitals, perineal, and inguinal regions. No predisposing conditions were found. Empiric wide broad antimicrobial therapy was administered (tigecycline, ertapenem, and metronidazole), and vasoactive support was needed. The patient immediately underwent wide surgical debridement (Figure 1), and VAC therapy was applied on the wound (Figure 2). Orchidectomy was not necessary. Hyperbaric oxygen therapy (20 minutes each at 2.4 ATA, 100% FiO_2_) was started after surgical toilette. During the sixth wound dressing, surgical colostomy was needed in order to limit the possibility of superinfection in anorectal area. The patient underwent 22 days of VAC therapy and 14 hyperbaric oxygen therapy sessions. 24 days after ICU admission, the patient was discharged to a high-dependency unit. Reconstructive surgery was later performed with good results.

### 2.2. Case  2

A 75-year-old man presenting dyspnoea, hypotension, and tachycardia was admitted to the emergency department of a peripheral hospital. An abscess was found in the right gluten with necrosis in the perianal and sacral areas, and rectal fistula. A computed tomography scan revealed gas formation on the pelvic cavity, on the right gluten and behind the corpora cavernous. Fournier's gangrene diagnosis was then made. The patient presented many risk and predisposing factors in his medical history: diabetes, chronic obstructive pulmonary disease, dilated cardiomiopathy, and chronic viral hepatitis. Before transfer to our ICU, empiric antimicrobial therapy with piperacillin/tazobactam, metronidazole, and vancomycin was initiated. After ICU admission, the patient immediately underwent surgical debridement. During surgical intervention, ileostomy was decided to isolate the rectal site of infection. VAC therapy was applied on the wound at the end of the surgical procedure, and hyperbaric oxygen therapy was started the next day. VAC device was removed after three days, and the patient was discharged to the postintensive ward the day after.

## 3. Discussion

The two cases reported were different in disease stadium and length of treatment. The most important feature in Fournier's gangrene management remains the timing of diagnosis; in the first case, the infection started one week before. This might cause the need for prolonged VAC therapy and numerous hyperbaric oxygen therapy sessions, in addition to wide surgical debridement. The early treatment performed in the second case permitted more conservative surgical intervention and lower duration of VAC therapy, hyperbaric oxygen therapy sessions, and ICU length of stay.

Despite appropriate treatment, mortality rates remain high, up to 67% [14], even if a recent article by Janane and coworkers reported, in a case series of 70 patients treated with both VAC therapy and hyperbaric oxygen therapy, a very low mortality rate (11.4%), failing also to confirm the predictive value of Fournier's gangrene severity score index [15]. Unfortunately, this report is available only in Spanish, thus, important details about this wide sample are unknown for many readers. Another case treated with both VAC devices hyperbaric oxygen therapy has been recently published by Assenza and coauthors [16].

As well as hyperbaric oxygen therapy [13], we suppose that the use of VAC devices can increase fibroblast migration and cell proliferation improving clinical outcome. We do not know if oxygen penetrates the plastic drape, but it might be reasonable that hyperbaric oxygen therapy can have more beneficial effects if combined with the reduction of edema and interstitial pressure, thanks to VAC dressing. When analyzing this kind of therapy in a relatively rare disease, the difficulty is that prospective randomized trials do not exist, and the limited number of patients in each study cannot allow strong statistical comparisons. Nevertheless, hyperbaric oxygen therapy has been demonstrated to reduce mortality rate in several case series [13]. Moreover, Cuccia and coworkers recently reported the good resolution of six cases treated with VAC therapy after surgical debridements [17]. In a recent prospective, comparative studies on 35 patients showed that patients treated with VAC device had a significantly lower mortality than patients in which daily antiseptic (polyhexanide) dressings were used [18].

Both of the patients presented in this paper underwent colostomy. The rationale for rectal diversion includes a decrease in the number of germs in the perineal region, improved wound healing, and local control of the infection [19]. Many surgeons believe that a colostomy is an integral part of management for patients requiring extensive debridement, especially if the infection arises in the anorectal region [6, 8]. Other authors recommend that a diverting colostomy should only be constructed in the presence of colorectal perforation, immunodepression, or if incontinence is present [20].

In addition, both patients were treated with Flexi-Seal Fecal Management System (Conva Tec USA, Skillman, New Jersey) for rectal diversion, which, for some authors, helps avoid the complications of performing a colostomy and subsequent reconstruction. The Flexi-Seal Fecal Management System consists of a soft silicone catheter with a retention balloon that is inserted into the rectum, a syringe for rectal irrigation, and a collection bag. It was designed to divert fecal matter in patients with diarrhea and skin ulcers in order to protect the wounds from contamination, especially in the intensive care unit setting [21].

## 4. Conclusions

Far from giving solid evidence regarding Fournier's gangrene treatment, our experience may suggest the consideration of the association of surgical treatment, VAC therapy, and sequential hyperbaric treatment for this life-threatening disease. Due to the lack of large studies, more medical literature, even in the form of case series, is needed to help clinicians.

## Figures and Tables

**Table 1:** Baseline and clinical characteristics of patients.

	Patient 1	Patient 2
Gender	Male	Male
Age (years)	63	75
BMI	23	29
SAPS II admission/discharge	47/24	42/22
SOFA admission/discharge	10/6	10/7
White cells count (N/mm^3^)	1,200	18,900
Platelet count (N/mm^3^)	54,000	331,000
Lactate dehydrogenase (U/l)	137	241
Creatine kinase (U/l)	61	20
Procalcitonin (ng/mL)	20.9	1.1
Duration of VAC therapy (days)	22	3
Duration of mechanical ventilation (days)	12	2
Length of stay in ICU (days)	23	4

SAPS: Simplified Acute Physiology Score; SOFA: Sequential Organ Failure Assessment Score; VAC: Vacuum-assisted closure.
